# Phenotype alteration causes long-term changes to the social strategies of victimised birds

**DOI:** 10.1038/s41598-023-29577-x

**Published:** 2023-02-10

**Authors:** Guiomar Liste, Inma Estevez

**Affiliations:** 1 Neiker, Animal Production Department, P.O. Box 46, 01080 Vitoria, Spain; 2 Department of Market Research, ESIC Business & Marketing School, Vía Ibérica 28, 50012 Zaragoza, Spain; 3 ESIC University, Av. de Valdenigrales, s/n, 28223 Pozuelo de Alarcón, Madrid, Spain; 4 Ikerbasque, Basque Foundation for Science, Alameda Urquijo 36-5 Plaza Bizkaia, 48011 Bilbao, Spain

**Keywords:** Zoology, Animal behaviour

## Abstract

Phenotype alterations can occur naturally during the life span of the domestic fowl. These alterations increase the risk to become a target of aggression and may cause a severe impact on the welfare of affected birds. We analysed the behavioural consequences of sequential phenotype alterations and their long-term effects within stable social groups of adult birds differing in group size. Phenotypically homogeneous groups, with 100% or 0% marked individuals, and heterogeneous groups, with 70%, 50% or 30% marked birds, were housed at constant density in groups of 10, 20 or 40. We applied sequential phenotype alterations to homogeneous groups (by marking or unmarking birds) and compared their behavioural response to heterogeneous groups considered controls. Results show that aggression was greatly affected by phenotype alteration but, unexpectedly, group size did not play any relevant role modulating social responses. Aggression was directed towards the first altered birds and was significantly higher than in control groups. Long term effects were detected, as victimized individuals failed to engage in aggression at any time and adapted their behaviour to minimize aggressive encounters (e.g. high perch use). Therefore, we provide evidence of long-lasting submissive strategies in stable groups of adult domestic fowl, highlighting the relevance of phenotype alteration on the social dynamics of affected birds. Phenotype alterations could help explain much of the targeted aggression observed in producing flocks which severely affects animal welfare.

## Introduction

Recent advances in the study of the social dynamics of the domestic fowl have shown that individuals that are phenotypically different from their conspecifics, due to a natural or artificial variation in feather colouration, are at a higher risk of receiving aggressive interactions from their flock mates^1,2^. The group pressure resulting from these interactions may affect the social, feeding or locomotory behaviours of altered individuals^3,4^ as a strategy to avoid interactions with flock-mates. Aggressive interactions in the domestic fowl are typically directed towards subordinates^5^ and sometimes linked to specific phenotypes^6^. Traditional studies on the social behaviour of the domestic fowl describe initial periods of intense aggression in small flocks until a social hierarchy is established^7^ and predict the number of interactions during hierarchy formation to increase with group size^8^. For a hierarchical social structure to be stable group members must recognize each other individually^7,9^ or through badges of status^6,9,10^. Badges of status are traits that signal fighting ability when individual recognition is not an option^11,12^, thus allowing dominant individuals to reduce the cost invested in aggression^13,14^. Familiarity based on appearance also plays a role on the ability to recognize group mates individually or based on badges of status, and on the frequency of aggressive interactions displayed^15^, which could also be dependent on group size^16^.

Experimental studies have shown that phenotype diversity leads to frequency-dependent targeted aggression in young domestic fowl, with more aggression directed towards those individuals showing the less common phenotype in the population^2,4,17^. Such studies have attempted to answer fundamental questions about the motivation behind aggressive interactions and considered their practical implications. Modern domestic fowl flocks are highly homogenous due to hybrid genetics (selection for health and performance traits) and management practices designed to accommodate the needs of the birds. Nevertheless, naturally occurring phenotype alterations may emerge repeatedly as a result of disease or injuries and are likely to affect different proportions of individuals within a group. Such alterations are more likely to take place as the flock ages and it is common to observe how individuals diverging from the average flock phenotype are targets for aggression (Estevez, personal observation). Repeated occurrences of social stress over time could induce physiological adaptation and habituation, with individuals learning to predict events and thus facilitating recovery^18^. Alternatively, it could induce chronic stress and the subsequent persistence of negative behavioural and physiological responses.

Phenotypic diversity might be a relevant factor affecting the welfare and performance of poultry, but it has received little scientific attention. Dennis et al.^2^ demonstrated its impact at the physiological, performance and behaviour level, and more recent studies^4,17,19,20^ are starting to explore this phenomenon that may be crucial for the understanding of the social dynamics of the domestic fowl. We have previously investigated the effects of phenotype alteration on young birds housed at different group sizes^4,17^. The current study is a follow up investigation designed to determine the impact of changing phenotype appearances in well-established social groups of adult laying hens. This study does not focus on the magnitude of aggressive interactions (already reported in^21^) but concentrates on immediate behavioural responses after phenotype alteration and the long-term consequences for affected birds.

The underlaying hypothesis was that the impact of phenotype alteration could be dependent on group size, type of alteration and time. Altered individuals were expected to face lower aggression in smaller groups since individual recognition or familiarity could be assumed in socially stable groups under 30 individuals^11,16,22,23^. Likewise, altered birds in small groups were also predicted to show fewer long-term behavioural changes. On the other hand, a more relevant impact of phenotype alteration was expected in larger groups of 40 birds, where individual recognition is challenging and social interactions may be determined by badges of status^11^. Social instability could also anticipate larger long-term behavioural changes for the victimized birds in bigger groups. Lastly, it was also predicted that the alteration (marking or unmarking) would not be the cause of behavioural changes, but the resulting difference from the average phenotype within the group. Therefore, responses were expected to be independent on whether diversity resulted from marking birds with an artificial phenotype or from removing an artificial mark applied as one day old chicks. The study of the behavioural responses to phenotype alteration contributes to a deeper understanding of social recognition mechanisms in the domestic fowl and its implications on social dynamics and animal welfare.

## Results

### Comparing behaviour of groups

At t0, prior to any phenotype manipulation, no significant differences in behaviour could be found between originally homogeneous (100% M and 100% U) and heterogeneous groups (30%, 50% and 70% M, respectively). On the other hand, group size did affect some of these behaviours (Supplementary Table S1) but no interactions between group size and phenotype treatment were statistically significant.

**Figure 1:**
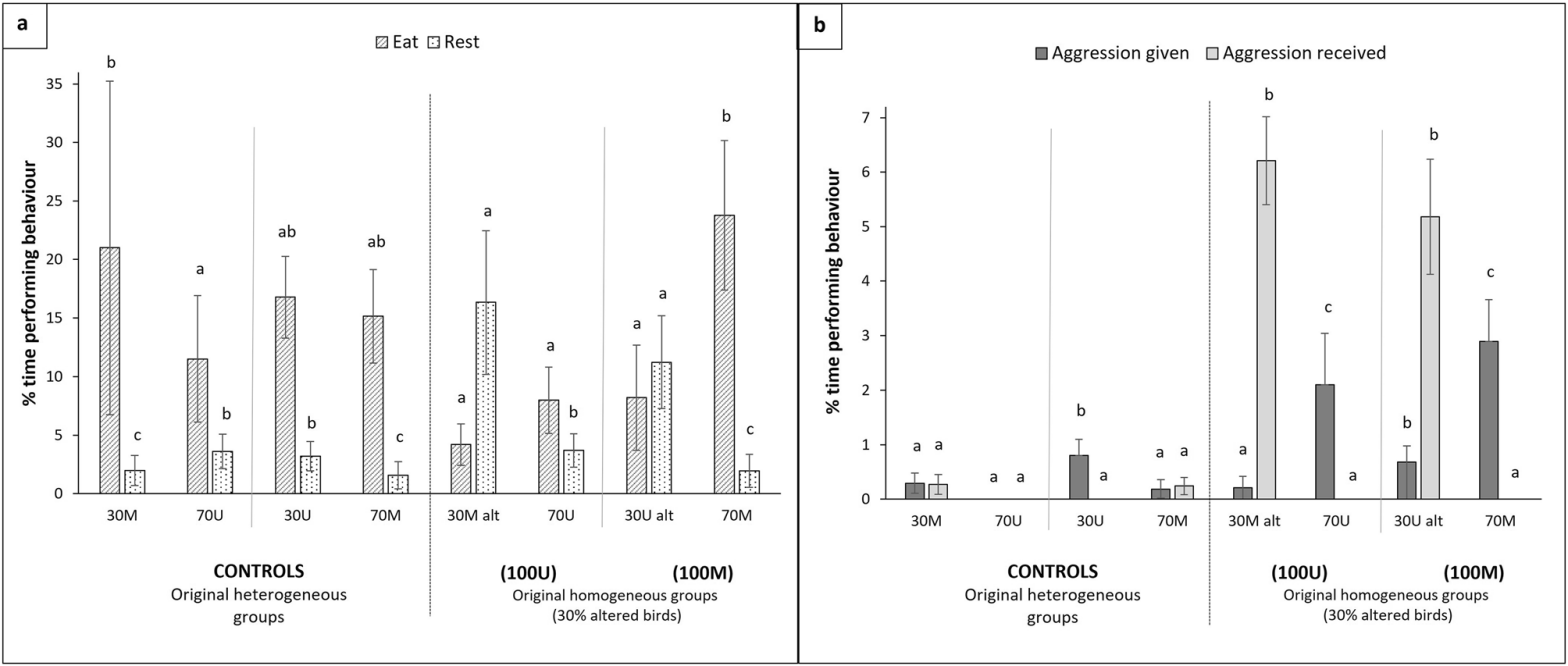
Effects of phenotype alteration to 30% of individuals on the behaviour of hens at 35–36 weeks of age (t1). M: marked; U: unmarked; alt: altered. Bars represent means ± standard errors. Different letters within the same behaviour indicate significant differences among groups at p < 0.05. (**a**) Effects on eating and resting behaviours. (**b**) Effects on aggression given and received.

Clear behavioural differences were detected after the 1st phenotype alteration at 34 weeks (t1). Altered groups of birds (30 M altered and 30U altered) showed a significant reduction of aggression given (H_7_ = 27.07, p < 0.001) and time spent eating (H_7_ = 15.96, p = 0.026) when compared to 30 M and 30U controls and their pen mates, and both groups showed higher aggression received (H_7_ = 56.76, p < 0.001) and time spent resting (H_7_ = 14.87, p = 0.038) as compared to their controls and pen mates (Fig. 1). Group size effects disappeared after this 1st alteration and there were no significant interactions to report.

**Figure 2:**
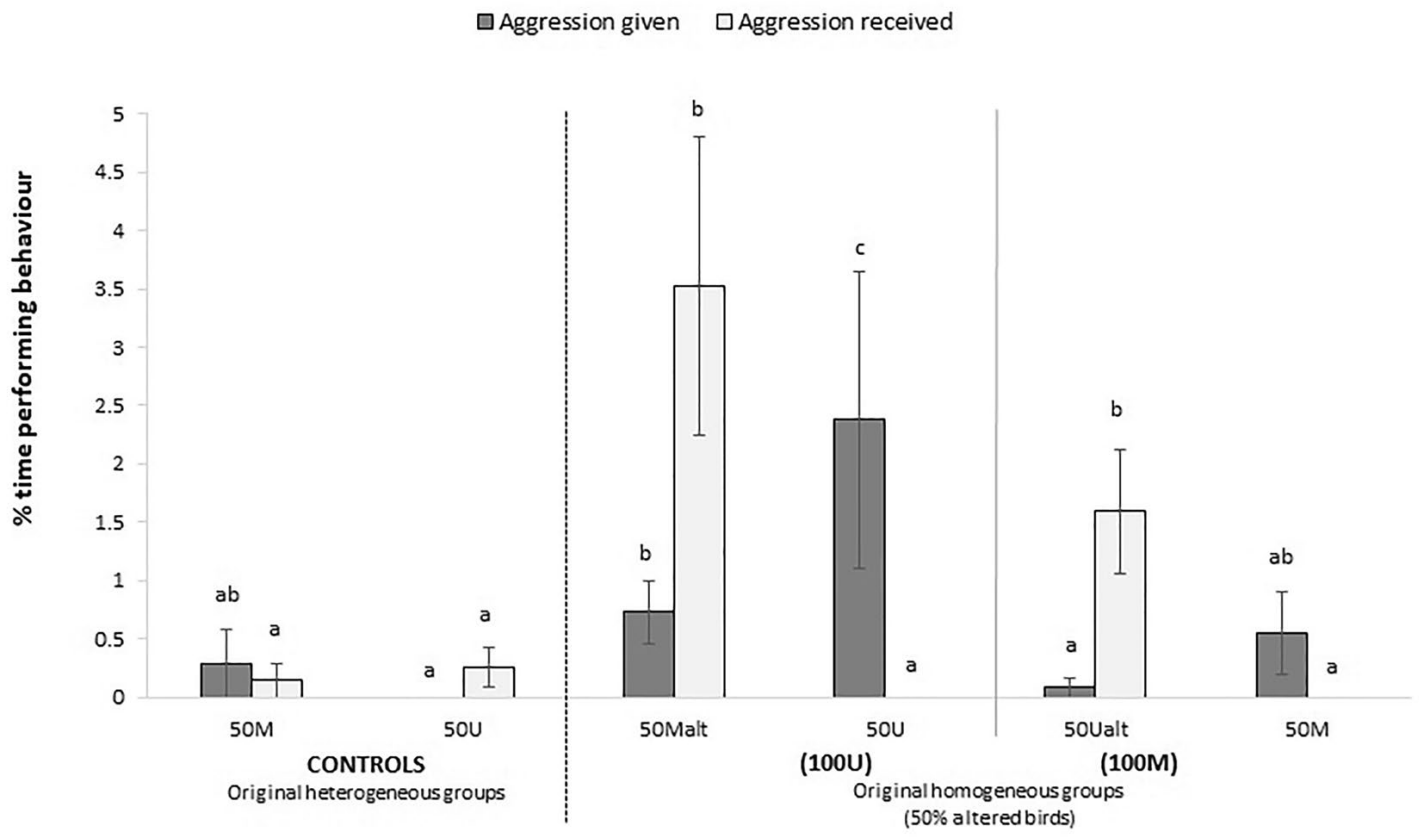
Effects of phenotype alteration to 50% of individuals on the behaviour of hens at 39–40 weeks of age (t2). M: marked; U: unmarked; alt: altered. Bars represent means ± standard errors. Different letters within the same behaviour indicate significant differences among groups at p < 0.05.

The 2nd phenotype alteration, creating groups with 50% individuals altered at 38 weeks (t2), only produced differences on the levels of aggression, with more aggression received by altered birds when compared to both their pen mates and controls (Fig. 2). Eat (H_2_ = 6.24, p = 0.044) and dust bath (H_2_ = 11.01, p = 0.004) were the only behaviours affected by group size (Supplementary Fig. S1). No significant interactions between group size and phenotype were observed.

**Figure 3:**
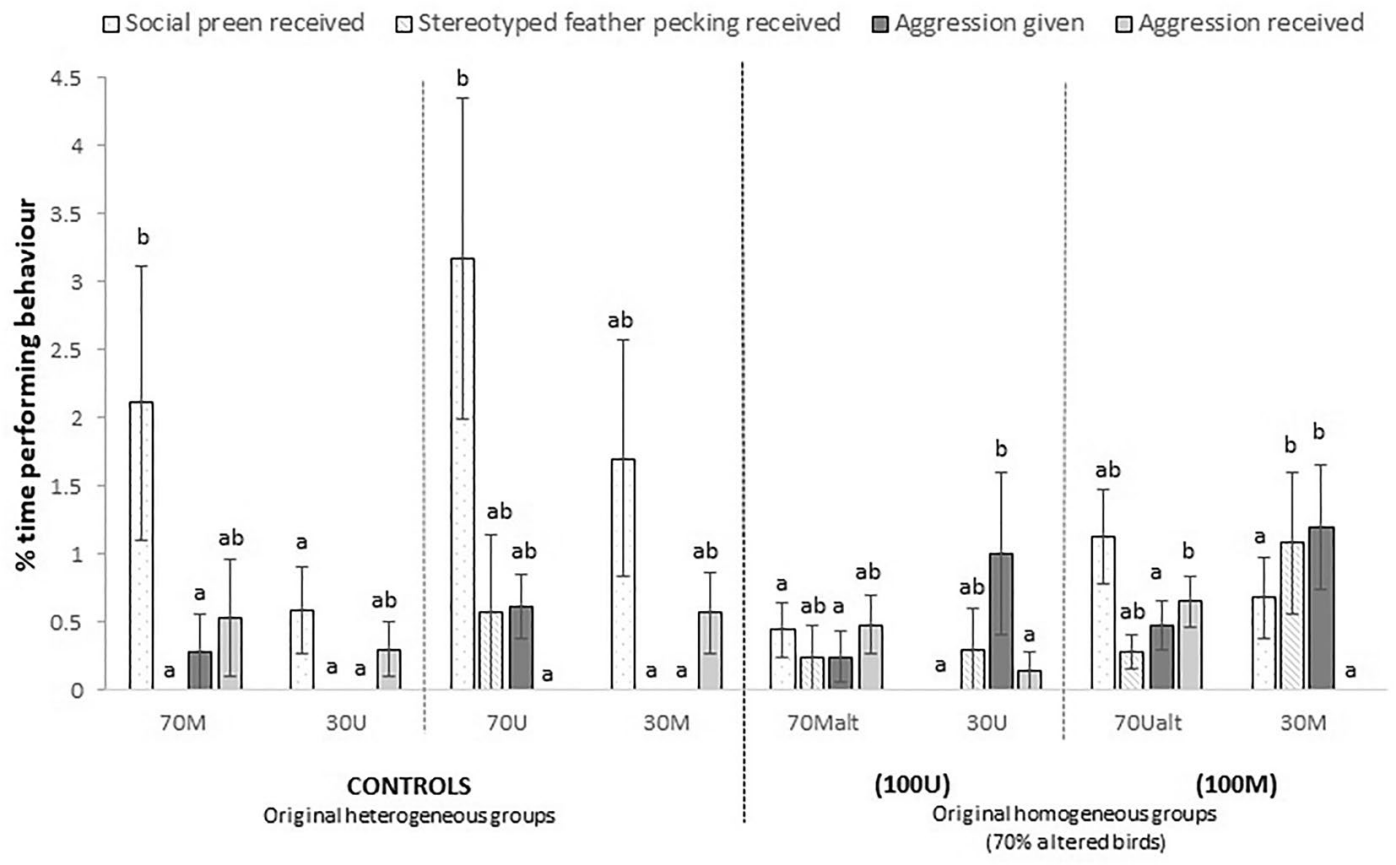
Effects of phenotype alteration to 70% of individuals on the behaviour of hens at 45–46 weeks of age (t3). M: marked; U: unmarked, alt: altered. Bars represent means ± standard errors. Different letters within the same behaviour indicate significant differences among groups at p < 0.05.

The last phenotype alteration at 44 weeks (t3) showed that aggression given was still high amongst the 30 M and 30U pen mates when compared to both their recently altered pen mates and controls (Fig. 3). Group size only affected the behaviour locomotion (F_2, 24_ = 3.92, p = 0.034), with hens housed in groups of 10 moving less than groups of 40 (2.31 ± 0.41 vs. 4.65 ± 0.84, p < 0.05) while groups of 20 were intermediate (3.65 ± 0.66). No interactions across main factors were detected.

### Comparing behaviour through time

These analyses aimed to depict the short and long-term effects of the phenotype alterations as they occurred, to better capture the ongoing changes on the laying hens being altered and the response from their never-altered group mates.

#### Never-altered individuals

Time affected the behaviour of the birds that, living within the groups ongoing sequential alterations, were never subjected to any phenotype alteration themselves: stand, exploratory peck, social preen received, stereotyped feather pecking given, stereotyped feather pecking received and aggression given (see ´never altered´ in Table 1). Group size only affected the locomotion of never-altered birds (F_2,42_ = 10.57, p < 0.001), with lower locomotion in birds from groups of 10 (3.42 ± 0.32) than those in groups of 20 (5.42 ± 0.51) and 40 (6.14 ± 0.58, p < 0.05 in both cases). No significant interactions between time, phenotype and group size were found to affect the behaviour of never-altered birds.

**Table 1:** Effects of time on the behaviour of birds living in groups subjected to sequential phenotype alteration (originally homogeneous 100M and 100U groups, altered by marking or unmarking).

Behaviour (% time budgets)	Observed birds (according to time of phenotype alteration)	Time of observation				Sig
		t0	t1	t2	t3	
Eat	Never altered	**15.93 ± 1.90**	15.89 ± 3.90	22.07 ± 2.15	15.71 ± 1.61	NS
	Altered at t1	*15.93* ± *1.90*^*a*^	**6.21 ± 2.41**^**b**^	7.40 ± 1.99^b^	12.11 ± 2.45^ab^	F_3,40_ = 7.15, p < 0.001
	Altered at t2	*15.93* ± *1.90*^*a*^	*15.89* ± *3.90*^*ab*^	**6.87 ± 2.69**^**b**^	14.37 ± 3.54^ab^	F_3,40_ = 4.50, p = 0.007
	Altered at t3	*15.93* ± *1.90*	*15.89* ± *3.90*	*22.07* ± *2.15*	**16.37 ± 1.97**	NS
Forage	Never altered	**10.99 ± 3.20**	7.01 ± 1.33	9.42 ± 1.78	6.08 ± 1.79	NS
	Altered at t1	*10.99* ± *3.20*^*a*^	**1.45 ± 0.42**^**b**^	5.17 ± 1.51^a^	6.88 ± 2.00^a^	F_3,40_ = 9.97, p < 0.001
	Altered at t2	*10.99* ± *3.20*	*7.01* ± *1.33*	**8.69 ± 2.69**	10.23 ± 2.62	NS
	Altered at t3	*10.99* ± *3.20*^*a*^	*7.01* ± *1.33*^*ab*^	*9.42* ± *1.78*^*a*^	**4.25 ± 0.80**^**b**^	F_3,40_ = 8.84, p = 0.006
Rest	Never altered	**2.13 ± 0.86**	3.90 ± 3.73	8.56 ± 3.70	7.11 ± 3.59	NS
	Altered at t1	*2.13* ± *0.86*^*a*^	**13.79 ± 3.59**^**b**^	2.71 ± 0.94^a^	3.44 ± 1.39^ab^	χ^2^_3_ = 3.99, p = 0.013
	Altered at t2	*2.13* ± *0.86*	*3.90* ± *3.73*	**2.39 ± 1.23**	2.03 ± 2.11	NS
	Altered at t3	*2.13* ± *0.86*	*3.90* ± *3.73*	*8.56* ± *3.70*	**2.36 ± 0.91**	NS
Stand	Never altered	**29.59 ± 3.63**^**a**^	19.92 ± 2.44^b^	18.78 ± 2.30^b^	25.27 ± 3.10^ab^	F_3,42_ = 4.66, p = 0.007
	Altered at t1	*29.59* ± *3.63*^*a*^	**30.80 ± 3.75**^**a**^	22.84 ± 2.77^a^	14.49 ± 1.76^b^	F_3,40_ = 9.05, p < 0.001
	Altered at t2	*29.59* ± *3.63*^*a*^	*19.92* ± *2.44*^*bc*^	**27.04 ± 3.23**^**ab**^	14.73 ± 1.76^c^	F_3,40_ = 9.87, p < 0.001
	Altered at t3	*29.59* ± *3.63*^*a*^	*19.92* ± *2.44*^*ab*^	*18.78* ± *2.30*^*ab*^	**16.96 ± 1.99**^**b**^	F_3,40_ = 3.59, p = 0.037
Locomotion	Never altered	**5.23 ± 0.94**	4.32 ± 0.71	4.79 ± 0.82	5.05 ± 1.21	NS
	Altered at t1	*5.23* ± *0.94*^*a*^	**1.87 ± 0.34**^**b**^	3.30 ± 0.58^ab^	2.93 ± 0.53^ab^	F_3,40_ = 5.57, p = 0.003
	Altered at t2	*5.23* ± *0.94*^*a*^	*4.32* ± *0.71*^*ab*^	**2.79 ± 0.46**^**b**^	3.12 ± 0.51^ab^	F_3,40_ = 3.71, p = 0.019
	Altered at t3	*5.23* ± *0.94*^*a*^	*4.32* ± *0.71*^*a*^	*4.79* ± *0.82*^*a*^	**2.13 ± 0.37**^**b**^	F_3,40_ = 7.75, p < 0.001
Exploratory peck	Never altered	**3.04 ± 0.65**^**a**^	1.49 ± 0.57^ab^	0.85 ± 0.58^b^	0.53 ± 0.15^b^	χ^2^_3_ = 7.93, p < 0.001
	Altered at t1	*3.04* ± *0.65*^*a*^	**1.60 ± 0.60**^**ab**^	1.28 ± 0.64^b^	1.51 ± 0.57^ab^	χ^2^_3_ = 4.28, p = 0.009
	Altered at t2	*3.04* ± *0.65*^*a*^	*1.49* ± *0.57*^*ab*^	**0.90 ± 0.48**^**b**^	0.76 ± 0.22^b^	χ^2^_3_ = 5.35, p = 0.003
	Altered at t3	*3.04* ± *0.65*^*a*^	*1.49* ± *0.57*^*ab*^	*0.85* ± *0.58*^*b*^	**0.74 ± 0.28**^**b**^	χ^2^_3_ = 7.56, p < 0.001
Comfort behaviours	Never altered	**10.37 ± 1.83**	13.60 ± 3.81	7.35 ± 1.79	9.67 ± 2.06	NS
	Altered at t1	*10.37* ± *1.83*	**5.98 ± 1.88**	12.72 ± 2.99	6.08 ± 1.73	NS
	Altered at t2	*10.37* ± *1.83*^*ab*^	*13.60* ± *3.81*^*a*^	**4.48 ± 1.11**^**b**^	5.40 ± 2.31^b^	F_3,40_ = 4.13, p = 0.011
	Altered at t3	*10.37* ± *1.83*^*ab*^	*13.60* ± *3.81*^*a*^	*7.35* ± *1.79*^*ab*^	**4.32 ± 1.05**^**b**^	F_3,40_ = 3.06, p = 0.039
Perch rest	Never altered	**7.94 ± 1.33**	13.12 ± 2.61	10.71 ± 3.19	15.23 ± 3.90	NS
	Altered at t1	*7.94* ± *1.33*^*a*^	**16.98 ± 2.84**^**b**^	26.41 ± 4.41^b^	24.20 ± 4.06^b^	F_3,40_ = 10.09, p < 0.001
	Altered at t2	*7.94* ± *1.33*^*a*^	*13.12* ± *2.61*^*ab*^	**21.39 ± 4.26**^**b**^	27.11 ± 5.39^b^	F_3,40_ = 7.78, p = 0.001
	Altered at t3	*7.94* ± *1.33*^*a*^	*13.12* ± *2.61*^*a*^	*10.71* ± *3.19*^*a*^	**24.21 ± 7.22**^**b**^	F_3,40_ = 7.09, p < 0.001
Social preen given	Never altered	**1.87 ± 0.44**	1.33 ± 0.57	2.79 ± 1.09	2.19 ± 0.98	NS
	Altered at t1	*1.87* ± *0.44*	**2.61 ± 0.99**	2.25 ± 1.06	2.19 ± 0.99	NS
	Altered at t2	*1.87* ± *0.44*	*1.33* ± *0.57*	**2.31 ± 1.09**	2.22 ± 0.99	NS
	Altered at t3	*1.87* ± *0.44*^*ab*^	*1.33* ± *0.57*^*ab*^	*2.79* ± *1.09*^*a*^	**1.00 ± 0.58**^**b**^	χ^2^_3_ = 3.78, p = 0.016
Social preen received	Never altered	**0.73 ± 0.15**^**a**^	0.57 ± 0.19^ab^	0.32 ± 0.20^b^	0.34 ± 0.17^ab^	χ^2^_3_ = 3.59, p = 0.020
	Altered at t1	*0.73* ± *0.15*^*a*^	**0.49 ± 0.19**^**ab**^	0.31 ± 0.20^b^	0.34 ± 0.17^ab^	χ^2^_3_ = 35.13, p < 0.001
	Altered at t2	*0.73* ± *0.15*	*0.57* ± *0.19*	**0.51 ± 0.21**	0.38 ± 0.22	NS
	Altered at t3	*0.73* ± *0.15*^*a*^	*0.57* ± *0.19*^*ab*^	*0.32* ± *0.20*^*b*^	**0.36 ± 0.23**^**ab**^	χ^2^_3_ = 1.93, p = 0.004
Stereotyped feather pecking given	Never altered	**1.80 ± 0.67**^**a**^	0.50 ± 0.25^ab^	1.19 ± 0.71^ab^	0.29 ± 0.17^b^	χ^2^_3_ = 2.89, p = 0.044
	Altered at t1	*1.80* ± *0.67*^*a*^	**0.41 ± 0.36**^**b**^	1.80 ± 0.70^a^	1.72 ± 0.80^ab^	χ^2^_3_ = 2.89, p = 0.044
	Altered at t2	*1.80* ± *0.67*^*a*^	*0.50* ± *0.25*^*b*^	**1.02 ± 0.63**^**ab**^	1.94 ± 0.78^a^	χ^2^_3_ = 2.94, p = 0.042
	Altered at t3	*1.80* ± *0.67*	*0.50* ± *0.25*	*1.19* ± *0.71*	**1.63 ± 0.77**	NS
Stereotyped feather pecking received	Never	**0.53 ± 0.19**^**a**^	0.00 ± 0.00^b^	0.14 ± 0.14^ab^	0.69 ± 0.31^a^	χ^2^_3_ = 4.93, p = 0.004
	Altered at t1	*0.53* ± *0.19*^*a*^	**0.00 ± 0.00**^**b**^	0.14 ± 0.14^ab^	0.69 ± 0.31^a^	χ^2^_3_ = 4.93, p = 0.004
	Altered at t2	*0.53* ± *0.19*^*a*^	*0.00* ± *0.00*^*b*^	**0.21 ± 0.21**^**b**^	0.14 ± 0.14^b^	χ^2^_3_ = 5.15, p = 0.003
	Altered at t3	*0.53* ± *0.19*^*a*^	*0.00* ± *0.00*^*b*^	*0.14* ± *0.14*^*ab*^	**0.70 ± 0.43**^**ab**^	χ^2^_3_ = 3.65, p = 0.018
Aggression given	Never	**0.04 ± 0.04**^**a**^	2.49 ± 0.60^b^	1.46 ± 0.68^ab^	1.10 ± 0.36^ab^	χ^2^_3_ = 7.79, p < 0.001
	Altered at t1	*0.04* ± *0.04*	**0.00 ± 0.00**	0.00 ± 0.00	0.01 ± 0.01	NS
	Altered at t2	*0.04* ± *0.04*^*a*^	*2.49* ± *0.60*^*b*^	**0.45 ± 0.21**^**a**^	0.55 ± 0.26^a^	χ^2^_3_ = 9.68, p < 0.0001
	Altered at t3	*0.04* ± *0.04*^*a*^	*2.49* ± *0.60*^*c*^	*1.46* ± *0.68*^*bc*^	**0.16 ± 0.16**^**ab**^	χ^2^_3_13.92, p < 0.001
Aggression received	Never	**0.10 ± 0.07**	0.00 ± 0.00	0.00 ± 0.00	0.01 ± 0.01	NS
	Altered at t1	*0.10* ± *0.07*^*a*^	**5.69 ± 0.66**^**c**^	2.62 ± 0.64^b^	0.55 ± 0.26^a^	χ^2^_3_ = 35.13, p < 0.001
	Altered at t2	*0.10* ± *0.07*^*a*^	*0.00* ± *0.00*^*a*^	**2.45 ± 1.25**^**b**^	0.28 ± 0.16^a^	χ^2^_3_ = 35.13, p < 0.001
	Altered at t3	*0.10* ± *0.07*^*a*^	*0.00* ± *0.00*^*a*^	*0.00* ± *0.00*^*a*^	**0.95 ± 0.34**^**b**^	χ^2^_3_ = 9.55, p < 0.001

Hens were grouped for the analyses according to their phenotype history as: never altered, altered at t1 (34 weeks of age), altered at t2 (38 weeks of age) and altered at t3 (44 weeks of age). Times of observation: t0 = 27–28 weeks (before any alterations occurred); t1 = 35–36 weeks (after 1st phenotype alteration); t2 = 39–40 weeks (after 2nd phenotype alteration); t3 = 45–46 weeks (after 3rd phenotype alteration). Data are presented as means ± standard errors or ilink estimates ± standard errors, according to the statistical analyses performed for each behaviour. Different letters within the same row indicate significant differences among times of observation at p < 0.05.

Bold values highlight the behaviour of recently altered birds at each time period (recently marked or unmarked).

Italic values represent the behaviour of birds yet to undergo phenotype alteration, to aid the visualization of the significant differences across time periods.

#### Individuals altered at t1

Birds altered at t1 showed drastic changes in their behaviour following their alteration: time eating, foraging, walking and all behaviours implying activity were ostensibly reduced, while resting was increased (see ‘altered at t1’, Table 1). In addition, the behaviours exploratory pecks (H_1_ = 5.15, p = 0.023) and stereotyped feather pecking given (H_1_ = 4.47, p = 0.035) were affected by the type of phenotype alteration, being generally higher for M birds as compared to U birds (2.68 ± 0.56 vs. 1.01 ± 0.33 and 1.49 ± 0.48 vs. 0.51 ± 0.32, for M or U, respectively). Regarding the effects of group size, eat (H_2_ = 6.33, p = 0.042), perch preen (F_2,40_ = 3.46, p = 0.041), exploratory peck (H_2_ = 8.01, p = 0.018) and time in nest (H_2_ = 6.07, p = 0.048) were affected (Supplementary Fig. S2a). Locomotion was also affected by the interaction between group size and time of observation (F_6,40_ = 5.23, p = 0.003; Supplementary Fig. S2b).

#### Individuals altered at t2

Birds altered at t2 showed behavioural changes similar to those occurring for birds altered at t1, with the exception of forage, rest, social preen given and social preen received (see ‘altered at t2’, Table 1). M birds performed less comfort behaviours (5.69 ± 1.42) than U ones (11.23 ± 2.07; H_1_ = 6.82, p = 0.011) but, other than this, effects were similar whether alterations were due to marking or unmarking. The effect of group size was detected by higher resting (F_2,40_ = 7.78, p = 0.001) in groups of 20 (20.71 ± 3.59) and 40 (20.64 ± 3.57, p < 0.05 in both cases) when compared to groups of 10 (8.95 ± 1.55). Standing behaviour was affected by the interaction between phenotype (M, U) and group size (F_2,40_ = 5.96, p = 0.005), with M birds standing significantly more time than U birds, but only for groups of 10 individuals (32.16 ± 6.08 vs. 18.67 ± 3.93).

#### Individuals altered at t3

Finally, most behaviours performed by birds altered at t3 were again significantly affected by the time of observation, with the exception of eat, rest and feather pecking given (see ‘altered at t3’, Table 1). Comfort behaviour was affected by phenotype, with M birds performing less comfort behaviours than U individuals (5.43 ± 1.04 vs. 10.23 ± 1.95; F_1,40_ = 6.76, p = 0.013). Standing behaviour of birds altered at t3 was also affected by group size (F_2,40_ = 3.59, p = 0.037) with birds standing more in groups of 10 (24.48 ± 2.65) than 40 (16.77 ± 1.82, p < 0.05), while groups of 20 were intermediate (23.28 ± 2.52). Forage behaviour showed an interaction between phenotype (M, U) and group size (F_2,40_ = 3.68, p = 0.021), with U birds foraging significantly more (14.30 ± 3.27) than M birds (4.86 ± 1.04), but only in groups of 40. Locomotion was also affected by the interacting effects of phenotype and group size (F_2,40_ = 5.94, p = 0.006; Supplementary Fig. S3).

## Discussion

We had previously investigated the effects of manipulating the phenotype of birds at day 1 of age, analysing its consequences during early rearing^4,17,19^. The current study goes a step further by looking at the behavioural consequences of manipulating the phenotype of adult laying hens that were maintained in socially stable groups up to 34 weeks of age. We measured the effects of different group sizes while considering the short- and long-term implications on altered and unaltered individuals.

The function of a robust hierarchy is to determine the relative relevance of individuals within a group so that dominant birds get priority of access to resources and consequently aggressive interactions are reduced^24^. Theoretically, the lower the number of individuals to establish a hierarchy, the lower the number of interactions required to achieve it^11^. Thus, it was hypothesised that the response to phenotype alterations would be dependent on group size, with altered birds in smaller groups facing fewer interactions since individual recognition, or familiarity, could be assumed in small, socially stable groups. On the contrary, the impact of altered phenotypes in larger groups that would challenge individual recognition was expected to be more relevant, since social interactions are likely to be determined by badges of status^11^. Likewise, recovery time was predicted to be shorter in smaller as compared to larger groups, since social turmoil would be expected to be resolved faster. The discussion of the results is presented according to the three main factors included in our analyses (group size, phenotype and time), which represent our research questions.

###  Group size

Considering the substantial increase in aggression observed during our study, it was startling to see that group size did not play a relevant role. The performance of some behaviours was reduced in smaller groups, with locomotion being lower through time in groups of 10 for never altered birds, and in general for all birds at t3. However, even though density remained constant across all group sizes, groups of 40 birds showed the highest mobility associated with the higher space efficiency of larger groups^4^. Our results lead to the highly relevant question of why the introduction of a relatively minor phenotype change in small stable social groups of laying hens, where hierarchies based on individual recognition are expected^9,11,16,22,23,25^, would produce considerable social turmoil. The unexpected lack of group size effects suggests that phenotype alteration had a strong enough effect to overrule any previous social organization. Birds in our study seemed unable to recognise their familiar pen mates with a new phenotype, even in the smallest groups. Thus, the domestic fowl must have evolved to pay close attention to relatively minor changes in phenotypic attributes of group members. Our results suggest that phenotype may have played an important role in the evolutionary history of the ancestors of the domestic fowl, perhaps as a mechanism to avoid competition for resources or prevent other damages (e.g., new parasites or diseases) from foreign populations that might outcompete the local ones. The high plasticity shown by young birds to adjust their behaviour to early changes^4,17,19^ contrasts with the extreme sensitivity to phenotype alterations demonstrated in the current study. This might be associated with early imprinting mechanisms that generate the image or archetype of group members^26,27^.

### Phenotype

The low frequency of aggressive interactions observed at t0, before the start of sequential phenotype alterations, indicated that all groups in the study were socially stable. However, the situation dramatically changed with the first phenotype alteration and aggressive interactions went up to twenty times higher in altered groups than in controls (Fig. 1b). Interactions were clearly unidirectional, from unaltered to altered birds within each group, irrespectively of the resulting phenotype produced by the alteration (M or U birds). The impact of altering the first 30% of the population’s phenotype also produced acute changes on most active behaviours such as eating, foraging, locomotion and exploration, which were clearly lower in altered birds. In addition, altered individuals rested up to five times more than their unaltered pen mates (Fig. 1a). Such sudden changes in behaviour prove the dramatic impact that phenotype alteration produces on victimized birds. Regarding the intensity and directionality of aggression, similar results were again observed in consecutive changes at t2 and t3. Aggression received by 50% altered birds (t2) was 5 to 25 times higher than their controls (Fig. 2) but once alteration reached 70% of individuals (t3) differences remained significant only between pen mates altered by unmarking. Aggression given was again higher at t3 in both the 30% U and 30% M birds that remained never altered, showing still up to 4 times more aggression given between pen mates (see Fig. 3).

Targeted aggression towards altered individuals was similar regarding directionality to the effects observed in pullets^4,17^. Nevertheless, the effects observed in the current study were much stronger and aggression got to a much higher level, particularly after the first change to 30% of the birds. Interestingly, phenotype alterations produced by both adding or removing a black mark had similar effects in directionality and intensity. Individuals undergoing phenotype alteration in our study were chosen at random, so we had equal possibilities of selecting dominant and subdominant-like birds in all groups. Thus, considering that the risk of victimization was not linked to prior social status, our results strongly suggest that the trigger for aggression was the unknown phenotype and not the existence of a mark. The impact of social stressors has been reported to have varied effects on food intake, depending on the type of stressor and the species^28^, but social stress by social defeat has been associated with less eating in defeated individuals^29,30^. It could be speculated that victimized individuals might be prevented from accessing feeders. However, our results point to attempts to reduce social exposure and minimize the risk of aggression received from conspecifics as the reason for the reduced feeding, reflected by the general reduction in all active behaviours. Our results demonstrate the relevance of the social context as evidenced by how a small change in appearance triggered a strong response, independent of group size. Hence, social dynamics in the domestic fowl appear to be far more complex than what was initially expected, and strongly linked to the social context^22,31^.

### Time

Comparisons through time offer the clearest evidence of the ongoing behavioural changes in our study. Results revealed that phenotype alteration did not only affect the frequency of aggressive interactions given and received but dramatically reduced feeding, foraging, walking and any behaviour that would imply activity of victimized birds, including comfort behaviours and feather pecking. For example, time eating was reduced by more than half and time spent foraging was reduced more than five times after t1 (Table 1). Furthermore, victimized birds increased most behaviours implying low activity, including resting and standing, which indicates that aggressive interactions do not depend on the invasion of personal space but rather on the level and directionality of active behaviours^32^. Thus, altered birds seemed to reduce their activity to the minimum to become ‘socially unnoticeable’ as a strategy to avoid aggressive interactions. It is interesting to note that victimized birds more than doubled the proportion of time perching during the entire observation period, while this was not the case for never altered birds (Table 1). This fact provides further support to the relevance of perches as a refuge^33,34^, since it is known that aggressive interactions will mainly occur at floor level while in highly active states^32^, whereas encounters are unlikely when perching^35^. As sequential alterations progressed, the amount of aggression received and the strategies which minimized becoming a target were still evident, although the magnitude of the response declined progressively (at t2 and t3). This fact could point to a process of habituation, but it is clearly noticeable that never altered birds were rarely receptors of aggressive interactions, while altered birds (especially those altered at t1) were never, or very rarely, givers of aggression. Furthermore, as more individuals became targets through changes introduced at t2 and t3, more birds responded by reducing activity to avoid receiving aggression, while fewer birds remained givers of aggression. This fact could better explain the reduction in the intensity of the interactions, instead of crediting habituation, especially when considering the cascade of behavioural consequences following suit to the targeted aggressive interactions.

Aggressive interactions are an important source of stress which may lead to changes in physiological stress indicators, reduced corporal condition^2,20,36^ and behavioural changes^21,37,38^. The shift in aggressive behaviour received from pen mates and the drastic behavioural changes observed in recently altered birds seem to suggest a negative stress effect^39^ that was most severe after the first alteration. Interestingly, aggression given by victimized birds was never increased by subsequent phenotypic alterations of their remaining group mates. These results would support the idea that, even though a reduction in the intensity of behavioural responses could be observed, there were long-lasting effects evidencing social defeat. Lastly, we could consider the effects observed in our study as a response to social mismatch. Phenotype matching is a basic behavioural process occurring in the postnatal development phase that serves animals to learn the phenotypes of their group-mates, creating a template to compare against new and unfamiliar phenotypes^40,41^. These templates are normally developed during the first weeks of age^41,42^ to assure the correct identification of familiar individuals in the surrounding environment, before individuals disperse. The strong response observed in our study could indicate that domestic fowl are very sensitive to phenotype matching and are likely to generate a strong response against individuals not matching their templates. The risk of social mismatch may be even higher in large commercial flocks, since any factors affecting feather condition could turn individuals into potential victims. Further attention should be given to the impact of imprinting-like mechanisms allowing the adaptation to social life conditions encountered throughout an individual’s life^26,27,43^, since these mechanisms represent a core system dedicated to the development of social behaviour models.

## Conclusions

Contrary to our expectations, group size did not play a relevant role modulating the behaviour of domestic fowl subjected to sequential phenotype alteration. Phenotype changes severely affected the behaviour of birds originally housed in socially stable groups, especially regarding aggression. Repetition somehow reduced the effects of phenotype alteration but victimized birds showed evidence of long-lasting behavioural effects due to the social challenge and the consequences of the associated negative stress. The current study sheds light into relevant aspects of social behaviour in the domestic fowl, such as the effects of exposure to repeated social stress, and contributes to our general understanding of social recognition mechanisms in captive animals. The results could also be useful to poultry producers as a means to understand aggression due to phenotype mismatching and to help them reduce the negative effects on the birds’ welfare.

## Methods

### Experimental facilities

The study took place at the experimental poultry facility in Neiker (Vitoria-Gasteiz, Spain). This facility has two lines of automatic drinkers and feeders as well as a computerized control system for light, ventilation and temperature. The barn was divided into 45 pens built with PVC piping and plastic netting. An opaque black plastic sheet was attached to the lower part of the netting to avoid visual contact between neighbouring groups of birds. Bedding was provided in the form of wood shavings (approx. 1.5 kg/m^2^) and birds were fed a commercial diet ad libitum according to their rearing phase. Feeder space was standardized to 4 cm/bird (round feeder) and one nipple drinker was provided per five birds. When birds reached 14 weeks of age, they were also provided with group nests (90 cm^2^ of nesting space per bird) and perches (15 cm of linear perch per bird) according to current European legislation (Council Directive 1999/74/EC). The lighting, ventilation and temperature regimes followed standard commercial practices.

### Animals and treatments

A total of 1050 beak-trimmed female 1-day-old Hy-line brown chicks (Hy-Line Brown) were obtained from Avigán Terralta S. A. (Tarragona, Spain). Birds arrived to the experimental facility and were randomly assigned to one of the 45 experimental pens in groups of 10, 20 or 40 (15 pens per treatment). All groups were kept at the same rearing density (8 birds/m^2^), therefore pen dimensions varied according to group size. Pens housing 10, 20 and 40 birds measured 0.75 × 1.78 m (1.25 m^2^), 1.00 × 2.50 m (2.5 m^2^) and 2.00 × 2.50 m (5.00 m^2^), respectively. Group size treatments were combined in a full factorial setup with a series of manipulations to the phenotype, where the birds’ appearance was artificially altered to achieve five different initial treatments: homogeneous groups with 100% marked individuals (100M), homogeneous groups with 100% unmarked individuals (100U), heterogeneous groups with 30% marked and 70% unmarked individuals (30M/70U), heterogeneous groups with 50% marked and 50% unmarked individuals (50M/50U) and heterogeneous groups with 70% marked and 30% unmarked individuals (70M/30U). Subsequently, for each group size and phenotype combination there were three replications.

The initial alteration of the birds’ phenotype was carried out at one day of age by placing a black mark with a non-toxic dyer on the back of the head of each bird, following previous experimental procedures^2^. Birds were remarked as needed at the end of each data collection week. In addition, all birds were identified with two white laminated paper tags including the pen number and the individual identity number (two-digit black numbers printed on both sides). The tags were fixed to the sides of the neck with plastic filaments injected through the skin^44^. More specific descriptions of materials and methods used at this initial stage of the experiment can be found in^4^. Animals were kept in these conditions up to the point of lay and we conducted experiments about the effects of group size and variable proportions of phenotypes on behaviour, production and space use^4,17,19^.

When animals entered into full production, sequential alterations in the appearance of the birds were introduced to the originally homogeneous groups (100M and 100U). The 1st phenotype alteration (t1) was applied to all birds at 34 weeks of age by marking or unmarking 30% of individuals from the homogeneous groups (100U and 100M) which resulted into 70U/30M and 70M/30U groups. At 38 weeks of age, a 2nd phenotype alteration (t2) was applied and an extra 20% of individuals from the originally homogeneous groups were marked or unmarked. These groups presented at that point a configuration of 50U/50M and 50M/50U. Finally, at 44 weeks of age, a 3rd and last phenotype alteration (t3) was applied to another extra 20% of individuals, and groups then presented appearances as 30U/70M and 30M/70U (Table 2). Original groups with heterogeneous phenotype compositions remained unchanged and served as controls.

**Table 2:** Experimental design showing phenotype treatments and their sequential alteration.

	t0 (up to 34 weeks)	t1 (34 weeks)	t2 (38 weeks)	t3 (44 weeks)
Original homogeneous groups	100M	30U/70M	50U/50M	70U/30M
	100U	30M/70U	50M/50U	70M/30U
Original heterogeneous groups: CONTROLS	30U/70M	** - - - -- - - - - - - - -- - - - - - - - - -- - - -- - - -- - - -- - - -- - - - →**		
	50M/50U	** - - - -- - - - - - - - -- - - - - - - - - -- - - -- - - -- - - -- - - -- - - - →**		
	30M/70U	** - - - -- - - - - - - - -- - - - - - - - - -- - - -- - - -- - - -- - - -- - - - →**		

*M* marked, *U* unmarked, *t0* initial treatments, *t1* 1st phenotype alteration, *t2* 2nd phenotype alteration, *t3* 3rd phenotype alteration.

### Data collection

Direct behavioural observations were carried out at 27–28 weeks of age (t0) prior to any changes in the original phenotype of the birds to set a baseline level of the behaviours studied. From there onward, 2-week observations were conducted after each phenotype alteration at weeks 35–36 (t1), 39–40 (t2) and 45–46(t3). Observations were conducted from 09:00 to 14:00 h. The behavioural categories were based on previous work and included the following: eat, drink, forage, rest, stand, locomotion, peck, stereotyped peck, comfort behaviours, perch preen, perch rest, dust bath, social preen given and received, aggression given and received, feather pecking given and received, stereotyped feather pecking given and received and in nest (see^4^ for a detailed description of behaviours). The frequency, type and directionality of aggressive interactions, as occurring in this experiment, have been reported in detail and discussed elsewhere^21^.

A customized version of The Chickitizer software^45^, considering the scaled dimensions of the three experimental pen sizes, was used to collect behavioural data. Regarding the initial period of observations at t0, all 45 pens were observed twice (one time per week of observation). Two minutes focal observations were conducted on six randomly chosen birds per pen, three belonging to each phenotype in the case of heterogenous groups. The behaviour of the focal birds was recorded at fixed intervals every 10 s. The same observation protocol was applied after the 1stphenotype alteration (t1) observing six birds per pen, three belonging to each different phenotype. All pens containing 70M/30U or 70U/30M individuals (36 pens) were observed, whether they belonged to the recently altered groups or to the controls. For the third set of observations (t2, after the 2nd phenotype alteration) all 27 pens containing 50M/50U individuals were observed. Six birds were observed in the case of the nine control pens (3 birds per phenotype). In the case of the 18 recently altered pens nine birds were observed, three belonging to each phenotype appearance treatment: never altered (t0), altered at 34 weeks (t1) and altered at 38 weeks (t2). For the last period of observation (t3, after the 3rd phenotype alteration) all 36 pens containing 70M/30U and 70U/30M individuals were observed again. Following a similar process, six birds were observed in the 18 control pens (3 birds per phenotype) and 12 birds were observed in the 18 recently altered pens, three belonging to each phenotype treatment: t0, t1, t2 and t3. Behavioural data was expressed as time budgets (% of scans observed performing each behaviour within the two minutes’ observation).

### Statistical analysis

Time budgets were calculated for each behaviour per bird and then averaged by time period and pen, which was considered the statistical unit. All analyses were performed using the SAS 9.3 software package^46^. Two types of analyses were performed.

#### Comparing behaviour of groups

First we aimed to analyse the changes occurring transversally at each time point through between-groups analyses, comparing the behaviour of birds in altered groups with those from control groups of similar phenotype composition. Hence, the behaviour of birds in 30M/70U and 30U/70M groups, resulting from alteration at t1 or t3 respectively, were compared to control birds from 30M/70U and 30U/70M as needed. Likewise, 50U/50M groups resulting from t2 were compared to 50U/50M controls. These analyses were planned to provide evidence about behavioural changes occurring due to phenotype instability but did not include consideration of time effects as group composition varied through time.

Generalized linear mixed models assuming a gamma distribution were built. For this first set of analyses, phenotype and group size were included as fixed effects and pen as random effect. The phenotypes considered were a combination of proportion (30/50/70) and marking (M/U), considering the appropriate combinations at each time point. Significant differences between treatments were examined using Tukey post-hoc comparisons. Results for the behavioural data fitting the gamma distribution (forage, stand, locomotion, perch rest, perch preen and comfort behaviours) are presented back as estimates ± standard errors provided by the ilink function as representations of central tendency. All other behavioural variables were rarely observed or presented very low frequencies. In this case, Kruskal Wallis tests were used to assess the effects of phenotype and group size. Planned posthoc Mann–Whitney tests with Bonferroni corrections were used to further investigate significant differences among groups. Results are presented as means ± standard errors because, due to the very low frequency of those behaviours, all other central tendency representations that could be considered more appropriate (median and interquartile ranges, for example) equalled zero in all cases.

#### Comparing behaviour thorough time

Secondly, we analysed the changes occurring longitudinally across time through within-group comparisons by assessing the evolution of birds from homogeneous groups (originally 100M and 100U) as their appearances were progressively altered through time. These analyses were planned to further examine the ongoing changes through time and to determine the prevalence of the effects after the phenotype alteration.

For this second set of analyses, similar models were built with the type of phenotype (M/U) and group size as fixed effects, time as repeated measure and pen as random effect. A compound symmetry matrix was used to account for repeated observations in time. Again, results for the behavioural data fitting the gamma distribution (eat, drink, forage, stand, locomotion, perch rest, perch preen and comfort behaviours) are presented back as estimates ± standard errors provided by the ilink function. Significant differences between treatments were also examined using Tukey post-hoc comparisons. Friedman’s tests were employed to assess the effects of time on the behavioural data that did not fit parametric assumptions. Additional information about our model can be found in Supplementary Table S2 (goodness of fit and estimates of pen as representation of random variability).

### Ethics statement

The study was approved by the Ethical Committee at Neiker and the Livestock Services at the Regional Government (Diputación Foral de Alava, permit number CEE_2010_002). All experiments were performed in accordance with the relevant guidelines and regulations as stated in the “Real Decreto 1201/2005” that regulates the protection of animals used for experimental and other scientific purposes in Spain. The study was carried out following the recommendations in the ARRIVE guidelines. The birds involved in the experiment were previously used in a study to determine the effects of group size and phenotype in young laying hens^4,11^. When the project was completed (at approximately 60 weeks of age), the birds were sold and processed at an EU-approved abattoir following commercial practices.

## Supplementary Information

Supplementary Information.

## Data Availability

All the data on which the conclusions of the paper are based are presented in the paper and the Supplementary Materials. Nevertheless, the dataset used and analysed during the current study is available from the corresponding authors on reasonable request.
