# Author Correction: Phenotype alteration causes long-term changes to the social strategies of victimised birds

**DOI:** 10.1038/s41598-023-37778-7

**Published:** 2023-07-04

**Authors:** Guiomar Liste, Inma Estevez

**Affiliations:** 1 Neiker, Animal Production Department, P.O. Box 46, 01080 Vitoria, Spain; 2 Department of Market Research, ESIC Business & Marketing School, Vía Ibérica 28, 50012 Zaragoza, Spain; 3 ESIC University, Av. de Valdenigrales, s/n, 28223 Pozuelo de Alarcón, Madrid, Spain; 4 Ikerbasque, Basque Foundation for Science, Alameda Urquijo 36‑5 Plaza Bizkaia, 48011 Bilbao, Spain

Correction to: *Scientific Reports* 10.1038/s41598-023-29577-x, published online 10 February 2023

The original version of this Article omitted affiliations for Guiomar Liste. The correct affiliations are listed below.

Neiker, Animal Production Department, P.O. Box 46, 01080, Vitoria, Spain.

Department of Market Research, ESIC Business & Marketing School. Vía Ibérica 28, 50012. Zaragoza, Spain.

ESIC University. Av. de Valdenigrales, s/n, 28223. Pozuelo de Alarcón, Madrid, Spain.

The original Article has been corrected.

