# Supplementary material for: Phenotype alteration causes long-term changes to the social strategies of victimised birds
Source: Sci Rep. 2023 Feb 10;13:2421. doi: 10.1038/s41598-023-29577-x (PMC9918478; doi:10.1038/s41598-023-29577-x)
Supplement: Supplementary file 1 — Supplementary Information. [file 41598_2023_29577_MOESM1_ESM.docx]

**Supplementary information**

**Fig S1.** Effects of group size on the behaviour of hens at 39-40 weeks (t2). Bars represent means ± standard errors. Different letters within the same behaviour indicate significant differences among group sizes at p<0.05.

**Fig S2.** Behaviour of birds altered at t1 (34 weeks of age). Data are presented as means ± standard errors or ilink estimates ± standard errors, according to the statistical analyses performed for each behaviour. **S2a.** Effects of group size (10, 20 and 40) on the behaviour of hens. Different letters indicate significant differences amongst group sizes at p<0.05. **S2b.** Effects of the interaction between group size (10, 20 and 40) and time of observation (t0 = 27-28 weeks, t1 = 35-36 weeks, t2 = 39-40 weeks and t3 = 45-46 weeks) on the proportion of time in locomotion. Different letters indicate significant differences at p<0.05 (lower cases for differences among times of observation and upper cases for differences among group sizes; lack of letters means no significant differences were found among times of observation or group size treatments).

**Fig S3**. Interaction effects on the behaviour of birds altered at 44 weeks of age (t3). Effects of the interaction between group size (10, 20 and 40) and phenotype (M = marked, U = unmarked) on the proportion of time in locomotion. Data are presented as ilink estimates ± standard errors. Different letters indicate significant differences at p<0.05 (lower cases for differences among group sizes and upper cases for differences between phenotypes; lack of letters means no differences were found among group sizes or type of phenotype).

**Table S1**. Effects of group size on the behaviour of laying hens at 27-28 weeks of age (t0), prior to any phenotype manipulation. Data are presented as means ± standard errors or ilink estimates ± standard errors, according to the statistical analyses performed for each behaviour (only statistically significant results are presented). Different letters within the same row indicate significant differences among group sizes at p≤0.05.

| **Behaviours**  (% time budgets) | **Group Size** | | | **Sig.** |
| --- | --- | --- | --- | --- |
|  | **10** | **20** | **40** |  |
| Exploratory pecks | 3.86±0.61^a^ | 3.02±0.84^ab^ | 2.02±0.39^b^ | H_2_=5.97, p=0.051 |
| Comfort behaviours | 9.98±1.54^ab^ | 5.86±0.90^a^ | 11.10±1.71^b^ | F_2,18_=4.91, p=0.020 |
| Perch preen | 3.96±1.16^a^ | 2.85±0.68^a^ | 1.13±0.61^b^ | H_2_=5.73, p=0.057 |
| Dust bath | 0.00±0.00^a^ | 2.02±0.95^b^ | 0.22±0.15^ab^ | H_2_=7.92, p=0.019 |
| Social preen received | 0.59±0.25^a^ | 1.92±0.67^b^ | 0.61±0.26^ab^ | H_2_=6.54, p=0.038 |

**Table S2.** Goodness of fit and model estimates (pen as covariance) for the GLMMs used to analyse the effects of group size, phenotype treatment and time on the behaviour of birds living in groups subjected to sequential phenotype alteration (originally homogeneous 100M and 100U groups, altered by marking or unmarking). Hens were grouped for the analyses according to their phenotype history as: never altered, altered at t1 (34 weeks of age), altered at t2 (38 weeks of age) and altered at t3 (44 weeks of age).

| Behaviour | Observed birds  (according to time of phenotype alteration) | Goodness of fit | | Covariance parameter: PEN | |
| --- | --- | --- | --- | --- | --- |
|  |  | **Pseudo-Likelihood** | **Chi^2^/DF** | **Estimate** | **SE** |
| Eat | Never altered | 108.85 | 0.45 | 0.0001 | 0.0427 |
|  | Altered at t1 | 101.10 | 0.58 | 0.0256 | 0.0698 |
|  | Altered at t2 | 103.45 | 0.56 | 0.0187 | 0.0549 |
|  | Altered at t3 | 112.49 | 0.46 | 0.0030 | 0.0795 |
| Drink | Never altered | 75.04 | 0.59 | 0.0187 | 0.1529 |
|  | Altered at t1 | 71.45 | 0.32 | 0.3035 | 0.1818 |
|  | Altered at t2 | 66.03 | 0.64 | 0.0011 | 0.1415 |
|  | Altered at t3 | 65.95 | 0.51 | 0.1377 | 0.2123 |
| Forage | Never altered | 145.66 | 0.65 | 0.0004 | 0.0667 |
|  | Altered at t1 | 115.80 | 0.79 | 0.0212 | 0.0813 |
|  | Altered at t2 | 138.58 | 0.80 | 0.1044 | 0.1227 |
|  | Altered at t3 | 126.97 | 0.51 | 0.0000 | 0.04765 |
| Stand | Never altered | 90.30 | 0.17 | 0.0423 | 0.0398 |
|  | Altered at t1 | 102.17 | 0.25 | 0.0103 | 0.0272 |
|  | Altered at t2 | 106.26 | 0.19 | 0.0620 | 0.0436 |
|  | Altered at t3 | 98.36 | 0.24 | 0.0048 | 0.0235 |
| Locomotion | Never altered | 101.41 | 0.36 | 0.0023 | 0.0217 |
|  | Altered at t1 | 79.28 | 0.21 | 0.0029 | 0.0210 |
|  | Altered at t2 | 105.90 | 0.26 | 0.0172 | 0.0396 |
|  | Altered at t3 | 101.47 | 0.27 | 0.0184 | 0.0371 |
| Perch Rest | Never altered | 92.68 | 0.31 | 0.1094 | 0.1012 |
|  | Altered at t1 | 110.83 | 0.29 | 0.04184 | 0.05185 |
|  | Altered at t2 | 89.74 | 0.20 | 0.0566 | 0.0588 |
|  | Altered at t3 | 86.82 | 0.20 | 0.1067 | 0.0757 |
| Perch Preen | Never altered | 83.19 | 0.56 | 0.6414 | 0.5431 |
|  | Altered at t1 | 99.39 | 0.58 | 0.0592 | 0.1367 |
|  | Altered at t2 | 92.33 | 0.86 | 0.3359 | 0.2846 |
|  | Altered at t3 | 84.55 | 0.79 | 0.0047 | 0.1499 |
| Comfort Behaviours | Never altered | 138.63 | 0.70 | 0.0031 | 0.0592 |
|  | Altered at t1 | 93.68 | 0.51 | 0.0012 | 0.0387 |
|  | Altered at t2 | 115.44 | 0.56 | 0.1286 | 0.1148 |
|  | Altered at t3 | 134.93 | 0.61 | 0.0301 | 0.1028 |
